# Effects of oral losartan administration on homeostasis of articular cartilage and bone in a rabbit model

**DOI:** 10.1016/j.bonr.2022.101526

**Published:** 2022-03-28

**Authors:** Zhenhan Deng, Xueqin Gao, Hajime Utsunomiya, Justin W. Arner, Joseph J. Ruzbarsky, Matthieu Huard, Sudheer Ravuri, Marc J. Philippon, Johnny Huard

**Affiliations:** a Department of Orthopaedic Surgery, McGovern Medical School, University of Texas Health Science Center at Houston, Houston, TX, USA; b Steadman Philippon Research Institute, Vail, CO, USA; c The Steadman Clinic, Aspen, CO, USA

**Keywords:** Losartan, Bone, Articular cartilage, Microfracture, Homeostasis, AngII, angiotensin II, AT1, angiotensin type 1, RAS, rennin-angiotensin system, RA, rheumatoid arthritis, AGTR2, angiotensin II receptor type 2, AGTR1, angiotensin II receptor type 1, SMAD7, mothers against decapentaplegic homolog 7, DMM, destabilization of the medial meniscus, pSMAD2/3, phosphorylated mothers against decapentaplegic homolog, ARRIVE, Animal Research: Reporting of In Vivo Experiments, Micro-CT, Micro-computer tomography, 2D, 2 dimension, 3D, 3 dimension, BV, bone volume, BV/TV, bone volume/total volume, Tb.N, trabecular number, Tb.Th, trabecular thickness, Tb.Sp, trabecular seperation, Ct.Th, cortical thickness, EDTA, ethylenediaminetetraacetic acid disodium dihydrate, H&E, Hematoxylin &eosin, Col1, collagen type I, Col3, collagen type 3, pTAK1, phosphorylated transforming growth factor β (TGF-β)-activated kinase 1, OCN, osteocalcin

## Abstract

**Background and aims:**

Previous work has shown that oral losartan can enhance microfracture-mediated cartilage repair in a rabbit osteochondral defect injury model. In this study, we aimed to determine whether oral losartan would have a detrimental effect on articular cartilage and bone homeostasis in the uninjured sides.

**Methods:**

New Zealand rabbits were divided into 4 groups including normal uninjured (Normal), contralateral uninjured side of osteochondral defect (Defect), osteochondral defect plus microfracture (Microfracture) and osteochondral defect plus microfracture and losartan oral administration (10 mg/kg/day) (Losartan). Rabbits underwent different surgeries and treatment and were sacrificed at 12 weeks. Both side of the normal group and uninjured side of treatment groups tibias were harvested for Micro-CT and histological analysis for cartilage and bone including H&E staining, Herovici's staining (bone and cartilage) Alcian blue and Safranin O staining (cartilage) as well as immunohistochemistry of losartan related signaling pathways molecules for both cartilage and bone.

**Results:**

Our results showed losartan oral treatment at 10 mg/kg/day slightly increase Alcian blue positive matrix as well as decrease collagen type 3 in articular cartilage while having no significant effect on articular cartilage structure, cellularity, and other matrix. Losartan treatment also did not affect angiotensin receptor type 1 (AGTR1), angiotensin receptor type 2 (AGTR2) and phosphorylated transforming factor β1 activated kinase 1 (pTAK1) expression level and pattern in the articular cartilage. Furthermore, losartan treatment did not affect microarchitecture of normal cancellous bone and cortical bone of tibias compared to normal and other groups. Losartan treatment slightly increased osteocalcin positive osteoblasts on the surface of cancellous bone and did not affect bone matrix collagen type 1 content and did not change AGTR1, AGTR2 and pTAK1 signal molecule expression.

**Conclusion:**

Oral losartan used as a microfracture augmentation therapeutic does not have significant effect on uninjured articular cartilage and bone based on our preclinical rabbit model. These results provided further evidence that the current regimen of using losartan as a microfracture augmentation therapeutic is safe with respect to bone and cartilage homeostasis and support clinical trials for its application in human cartilage repair.

## 1 Introduction

Losartan is one of the angiotensin II (AngII) receptor blockers (ARBs), also known as angiotensin type 1 (AT1) receptor antagonists, a group of anti-hypertensive drugs that are widely used for blood pressure control (Keating, 2009). They modulate the rennin-angiotensin system (RAS) by blocking the activation of AT1 receptors, thus preventing binding with AngII and decreasing blood pressure (McDonald et al., 2005). Recent studies have suggested that losartan has a broader therapeutic potential for treatment of other diseases, such as Marfan syndrome, chronic kidney disease, and nonalcoholic fatty liver disease (Chiu et al., 2013; Ravera et al., 2006; Vos et al., 2018).

Oral administration of losartan has also been shown to have beneficial effects in the musculoskeletal system such as in rheumatoid arthritis (RA) via up-regulation of angiotensin II receptor type 2 (AGT2R) and down-regulation of angiotensin II receptor type 1 (AGTR1) (Wang et al., 2013). A dose of 6 mg/kg was found also to prevent bone trabecular thinning induced by ovariectomy in rats by preventing the typical reduction in bone microcirculation while also improving fracture healing (Rajkumar et al., 2013). Administration of high dose (300 mg/kg/day) or clinically relevant dose of losartan (10 mg/Kg/day) immediately after muscle injury effectively improves muscle healing via decreasing myostatin and increasing follistatin expression (Kobayashi et al., 2013). Losartan treatment has also been shown to improve the beneficial effect of muscle-derived stem cells on muscle healing after injury by reducing scar formation and increasing muscle regeneration through upregulating mothers against decapentaplegic homolog 7 (SMAD7) and myoblast differentiation 1 (MyoD) expression (Bedair et al., 2008; Kobayashi et al., 2016). Finally, losartan treatment has been shown to attenuate the progression of articular cartilage degeneration in a mouse model of osteoarthritis (OA) induced by destabilization of the medial meniscus (DMM) by down-regulation of pSMAD2/3 (Chen et al., 2015a).

Microfracture is a relatively simple, minimally invasive, and cost-effective technique to treat focal articular cartilage defects (Steadman et al., 2002; Steadman et al., 2001). However, microfracture has been shown to lead to fibrocartilage rather than normal hyaline cartilage regeneration (Dai et al., 2014; Steadman et al., 2002; Steadman et al., 2010; Truong et al., 2014; Xu et al., 2015). Previous work has shown that oral losartan (10 mg/kg/day) combined with microfracture enhances cartilage repair quality by increasing hyaline cartilage in a rabbit osteochondral defect injury model when compared with microfracture alone (Utsunomiya et al., 2020). Losartan treatment was also shown to increase CD31^+^ cells and decrease CD45^+^ cells in bone marrow concentrate (Nakama et al., 2020). Moreover, a recently published study has demonstrated that an optimal dose of intra-articular injection of losartan (1 mg/knee) enhanced microfracture mediated cartilage repair without causing detrimental effects when injected into contralateral normal uninjured cartilage, however, higher dosages (100 mg/kg/knee) caused normal cartilage degeneration (Logan et al., 2021). Since the current study group has several ongoing clinical trials (NCT05025956, NCT04815902, NCT04212650) utilizing losartan for augmentation of cartilage and other types of tissue repair, the aim of this study was to determine whether oral losartan would affect the homeostasis of normal cartilage and bone when used as microfracture augmentation treatment in a rabbit model. This study will help evaluate the safety of losartan on human uninjured, native cartilage and bone while undergoing microfracture augmentation treatments.

## 2 Materials and methods

### 2.1 Animal use ethics

This study followed the National Institutes of Health guidelines for the care and use of Laboratory animals (NIH Publications No. 8023, revised 1978) and is compliant with ARRIVE guidelines. Before initiation of this study, institutional ethical review board evaluated and approved this animal study. New Zealand rabbits approximately 4 months of age (3.0 ± 0.2 kg) were purchased from Charles River Laboratories (Wilmington, MA) and housed at Center for Laboratory Animal Medicine and Care at the University of Texas Health Science Center at Houston and maintained according to approved protocols (AWC 17-0022).

### 2.2 Rabbit treatment

To investigate whether losartan treatment will affect uninjured cartilage and bone homeostasis, we utilized the contralateral side uninjured tibia of different treatment groups of rabbits harvested at the time of sacrifice from our previous published study (Utsunomiya et al., 2020). The previous study focused on the effect of losartan on injured cartilage repair mediated by microfracture by analyzing injured cartilage while the current study investigated its effect on normal contralateral side of uninjured cartilage and bone of tibia to save animal life. Rabbits were randomly divided into the following treatment groups (*N* = 7/group): osteochondral defect only group (Defect); osteochondral defect plus microfracture group (Microfracture) and osteochondral defect plus microfracture plus oral losartan group (Losartan). In brief, an osteochondral defect of 5 mm in diameter and 2 mm depth was created in the patellar groove of the knee joint in each treatment group rabbit (Defect). For the Microfracture and Losartan groups, after creation of osteochondral defect, five microfracture holes were made in each defect using a 0.7 mm diameter burr with 2 mm depth as previously described (Utsunomiya et al., 2020). This microfracture procedure allows for bone marrow and mesenchymal stem cells from the subchondral bone and bone marrow to migrate into the injured cartilage defect area and served as source of cartilage repair stem cells. The contralateral sides of each group that were not operated on were utilized for this study. For the rabbits in the Losartan group, a dose of 10 mg/kg/day of losartan was administrated using oral feed by veterinary staff daily from day 1 after surgery for 12 weeks. Losartan tablets were grounded and mixed with food treat (mixture of shredded carrots, apples, and cabbage) and feed rabbits to ensure complete ingestion. After 12 weeks, all rabbits were sacrificed, injured side distal femur with osteochondral defect (used for previous publication) (Utsunomiya et al., 2020) and uninjured side tibia (used for this paper) were harvested for Micro-CT and histology. Additional 3 rabbits were used as normal uninjured control (Normal). Both left and right tibia were harvested for Micro-CT and histology.

### 2.3 Micro-CT scanning and analysis for tibial cancellous bone and cortical bone tissues

Rabbit tibias were fixed in neutral buffered formalin for 1 week and then underwent Micro-CT scan using Viva CT 40 (SCANCO Medical). Micro-CT scanning was performed using 38 μm resolution (voxel size), 70 kVP and 112 μA X-ray energy scanning parameters to evaluate the epiphysis and metaphysis cancellous bone, and cortical bone of the tibias. After obtaining 2D image slices, the view of interest was uniformly delineated. The analysis of epiphysis was performed on a region starting 10 slices below the articular cartilage and extending 40 slices towards the proximal tibia. The analysis of metaphysis cancellous bone was performed on a region that was 10 slices below the growth plate and extended 120 slices towards the distal tibia. The tibial cortical bone analysis was performed on a region starting 400 slices from the end of the growth plate and extending 100 slices towards the distal tibia (Farooq et al., 2017). Representative images of the top view of each portion of the proximal tibia were saved as 3D images after 3D reconstructions. The bone microarchitecture parameters for each part of the trabecular bone included bone volume (BV), bone volume/total volume (BV/TV), bone volume (BV) density, trabecular number (Tb.N), trabecular thickness (Tb.Th) and trabecular separation (Tb.Sp) were generated automatically by Micro-CT software (Yao et al., 2013) using Gauss = 0.8, Sigma = 1, and threshold of 230 for cancellous bone. For the tibial cortical bone, we used Gauss = 0.8, Sigma = 1 and 280 threshold parameters. Cortical thickness (Ct.Th) and cortical bone volume density were generated automatically by Micro-CT software and used to represent cortical bone microarchitecture.

### 2.4 Histology analysis for tibia plateau cartilage and cancellous bone of tibia

After Micro-CT scanning, the entire tibia including the tibia plateau cartilage was decalcified using 10% ethylenediaminetetraacetic acid disodium dihydrate (EDTA) plus 1% sodium hydroxide for 4 weeks and followed by 5% formic acid for another 2 weeks. Tissues were then processed in gradient alcohol, cleared in xylene, infiltrated in paraffin, and then paraffin embedded. Sections were cut at 5-μm thickness using a microtome to the comparable level including both bone and cartilage. Section slides were deparaffinized in xylene, and then hydrated via ethanol gradient to water. H&E staining was performed to reveal general morphology of bone and cartilage using Hematoxylin extra-strength and Eosin Y-Alcoholic reagents (ANATECH LTD). Herovici's staining was used to differentiate collagen type 1 and 3 using protocols as previously described (Gao et al., 2016; Gao et al., 2014; Turner et al., 2013). For bone tissues, red collagen type I (Col1) area percentage was analyzed based on 100× magnification. For the cartilage, blue color that represents collagen type 3 (Col 3) was analyzed based on 100× images. In addition, specific staining for cartilage was also performed. Alcian blue staining was performed to detect hyaluronic acid matrix, and sulfate mucin using IHC world protocol (http://www.ihcworld.com/_protocols/special_stains/alcian_blue.htm). Safranin-O/fast green staining was also performed using IHC World protocol modified by extending the Safranin O step to 30 min (http://www.ihcworld.com/_protocols/special_stains/safranin_o.htm) to detect proteoglycan and glycosaminoglycan matrices. All reagents were purchased from Sigma. Cartilage morphology was scored using the Osteoarthritis Research Society International (OARSI) rabbit cartilage osteoarthritis histopathology grading system based on Safranin O staining (Laverty et al., 2010).

### 2.5 Immunohistochemistry of cartilage and bone

Immunohistochemistry of AGTR1, AGTR2, phosphorylated transforming growth factor β (TGF-β)-activated kinase 1 (pTAK1) and osteocalcin (OCN) were conducted using a similar protocol as previously described (Deng et al., 2019; Gao et al., 2019). Section slides were deparaffinized and rehydrated to H2O. Then the section slides were first incubated in Tris-EDTA buffer (pH = 8.0) containing proteinase K (10 μg/ml) for 20 min in a 37 °C water bath for antigen retrieval followed by immunohistochemistry. Briefly, after antigen retrieval, the section slides were washed in phosphate-buffered saline (PBS) three times and blocked with 5% donkey serum for 1 h at room temperature. Then, the slides were incubated with rabbit anti-AGTR1 (NBP1-77078SS, Novus Biologicals, 1:400 dilution), rabbit anti-AGTR2 (NBP1-77368, Novus Biologicals, 1:200 dilution) and rabbit anti-pTAK1 (Catalog # BS-3439R, ThermoFisher Scientific, 1:300 dilution) and mouse anti-osteocalcin (sc-365,797, Santa Cruz Biotechnology, 1:50 dilution) in blocking buffer at 4 °C overnight. On second day, slides were washed in PBS for 3 times and endogenous peroxidase was inactivated with 0.5% hydrogen peroxide (H_2_O_2,_ Sigma-Aldrich) in PBS for 30 min. After the PBS wash, section slides were further incubated with the biotinylated goat-anti-rabbit secondary antibody (BA 1000, Vector Laboratories, Burlingame, CA, USA, 1:300 dilution) for AGTR1, AGTR2, pATK1 or biotinylated horse anti-mouse secondary antibody (BA-2000, Vector Laboratories, 1:300 dilution) for OCN for 2 h at room temperature. Then slides were rinsed with PBS and incubated with VECTASTAIN® Elite ABC-HRP Kit, Peroxidase (Standard) (PK-6100, Vector Laboratories) for 2 h at room temperature. Subsequently, the DAB color reaction (SK-4100, Vector Laboratories) kit was used to reveal specific antigen-positive cells. Hematoxylin QS (H3404, Vector Laboratories) counterstaining was performed to reveal nuclei. Finally, slides were dehydrated through gradient alcohol, cleared with xylene, and mounted in Cytoseal medium.

### 2.6 Histomorphometry analysis

Images of cartilage were captured at different magnification for the different histology staining to include the entire articular cartilaginous surface of the tibia plateau using NIKON ECLIPE Ni upright microscope. For the bone tissues, both subchondral bone and the epiphyseal bone were analyzed for different targets. 5–6 representative images of the different bone tissues were captured for analysis. NIKON NIS software was used to quantify red Col1 in Herovici's staining for bone, blue Col3 in Herovici's staining for cartilage, and the blue cartilage matrix in Alcian blue staining. The positive area percentage of AGTR1, AGTR2 was also analyzed using NIKON NIS software to identify the brown pixels percentage. The positive area percentage of these different staining was used to represent the expression level of the above matrices or genes. For the pTAK1 staining in the articular cartilage, the positive cells were counted in all images (7–15 images) that include the entire articular cartilage area (above tide marker) using Image J. Furthermore, the cartilage area was measured at the same time and normalized to 200× field and expressed as cell number/ 200× field cartilage area.

For the quantification of pTAK1, OCN of bone tissues parts, we counted positive cells on bone surface of epiphysis or subchondral bone with Image J and at the same time measured bone area and perimeter. For OCN and pTAK1 on epiphysis bone, we expressed values as positive cells/mm bone surface. For OCN ^+^cells on subchondral bone, we expressed values as positive cells/200 × field bone area (normalized to 200× field).

### 2.7 Statistical analysis

Analysis of variance (ANOVA) was used to compare parameters of the 4 groups followed by Tukey's post-hoc comparison using Graphpad Prism 9 software. *P* < 0.05 was considered statistically significant. All values are expressed as the mean ± SD.

## 3 Results

### 3.1 Effect of losartan treatment on articular cartilage of the tibia plateau

**Fig. 1:**
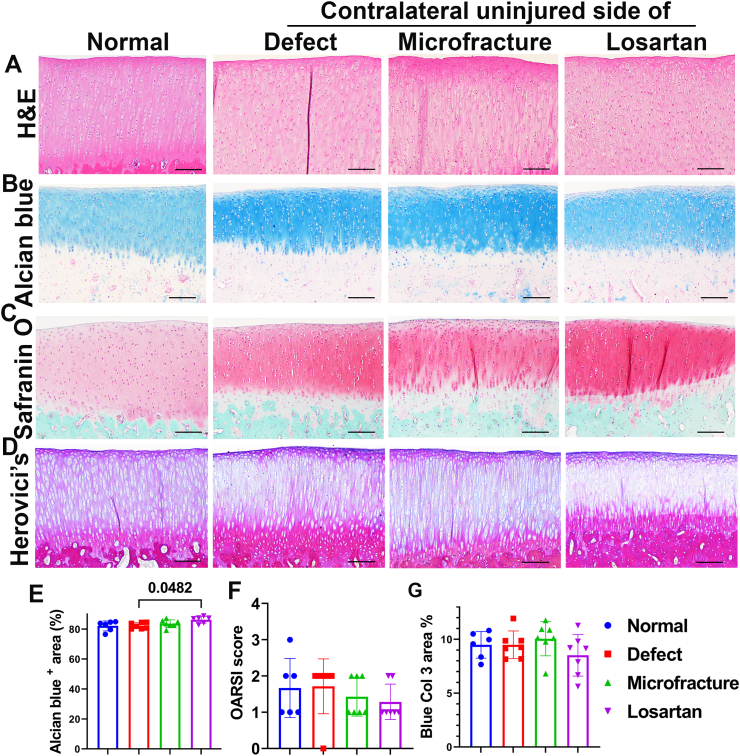
Histology assessment of tibial articular cartilage. (A) H&E staining showed no pathological changes in the articular cartilage of all groups. (B and E) Alcian blue staining and quantification. The Losartan groups showed a slightly increased level when compared to any other group, and a significant increase in the percentage area of blue matrix compared to the defect group. *P* value is shown between group bars. (C and F) Safranin O staining demonstrating GAG of articular cartilage (orange/red) in each of the 4 groups as well as OARSI score. Overall, the Losartan, Defect, Microfracture groups have relatively stronger staining than the Normal group. No statistical difference between the Losartan group and the other groups in term of OARSI score. (D and G) Herovici's staining and blue Col 3 quantification. Cartilage showed mixed blue and red matrix in all groups. The area percentage of blue Col3 was relatively lower in the Losartan group than other groups. No statistical differences were found between Losartan group and other groups. All images are 100×. Scale bars = 200 μm for all images.

This is a follow-up work of a previously published study which demonstrated that losartan at 10 mg/Kg/day promoted microfracture-mediated cartilage repair (Utsunomiya et al., 2020). To test whether this dosage had any detrimental or beneficial effect, on the uninjured cartilage, the current study was conducted. H&E staining showed no differences in the general morphology, cellularity, and structure of the articular cartilage between the Losartan, Normal, Defect and Microfracture groups (Fig. 1A). Alcian blue staining demonstrated the staining intensity of the Losartan, Defect and Microfracture groups were similar or stronger than the Normal group. The Losartan group showed a slight but significant increase in Alcian blue positive matrix percentage compared to the Defect group (ANOVA, *P* < 0.05). No statistical differences between any other groups were identified (Fig. 1B, E). Safranin O staining demonstrated that all groups had normal cartilage structure with slight variation of staining intensity within and between groups. The Losartan group showed overall relatively similar intensity of proteoglycan and glycosaminoglycan (orange red) content compared to the other groups (Fig. 1C). Using the OARSI scoring system evaluation criteria for rabbit osteoarthritis, the perichondrium of the majority of specimens stained lighter than other layers of cartilage in all groups which scored 1 for Safranin O staining. Very minor cartilage surface fissures were also observed in all groups (not due to section issues) which resulted in a score more than zero overall for all groups. No other pathological changes were observed in any groups. No statistical differences of OARSI histology scores were revealed between the Losartan group and any other groups (Fig. 1F). Herovici's staining of the cartilage layer revealed a mixture of red Col 1 and blue Col 3 matrix in all groups (Fig. 1.D). The area percentage of blue Col 3 was relative lower (fibrosis indicator) in the Losartan group compared to other groups, but not reach statistical difference (Fig. 1G, *P* = 0.2732 versus Microfracture group).

### 3.2 Effect of losartan on Angiotensin II receptors and pTAK1 expression in articular cartilage of tibia plateau

**Fig. 2:**
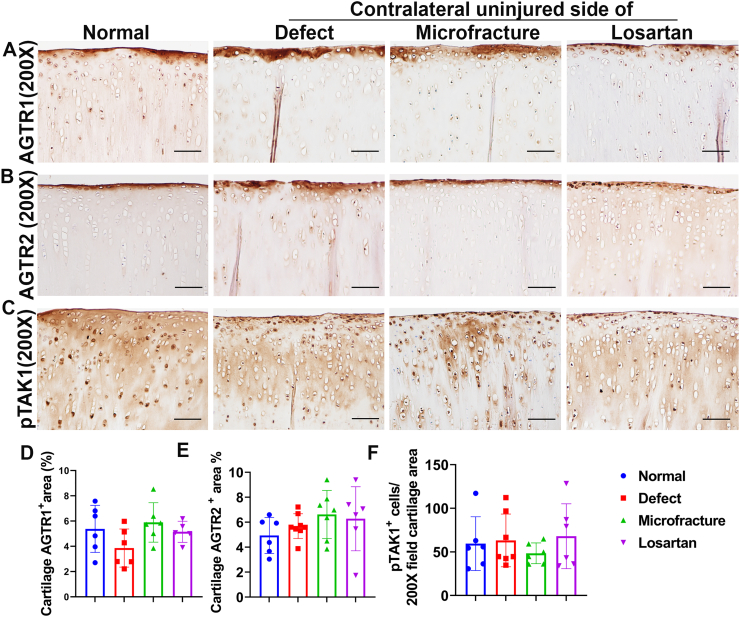
Immunohistochemistry of signal molecules in articular cartilage of tibia plateau. (A and D) AGTR1 expression and quantification. AGTR1 is mainly expressed in the perichondrium of all groups. Quantification showed no statistical differences between the Losartan group and the other groups. (B and E) AGTR2 expression and quantification. AGTR2 is mainly expressed in the perichondrium of articular cartilage in all groups. No statistical difference was found between the Losartan group and the other groups. (C and F) pTAK1 expression in articular cartilage and quantification. Positive cells are shown as brown nuclear staining across all cartilage layers. No statistical differences were found in the pTAK1 positive cell numbers between the Losartan group and the other groups. All images are 200×. Scale bars = 100 μm.

Immunohistochemical staining results demonstrate that AGTR1 receptor is mainly expressed in the perichondrium layer of cartilage as showed by a brown color. No difference of expression pattern was found between the Losartan group and the other groups (Fig. 2A). Quantification of AGTR1 positive area percentage showed no statistical difference between the Losartan group and the other groups (Fig. 2D). Similarly, the AGTR2 receptor was also found to be expressed in the perichondrium of cartilage in all groups (Fig. 2B). Quantification indicated that the AGTR2-positive area percentage showed no statistical differences between the Losartan group and the other groups (Fig. 2E). Furthermore, the pTAK1 expression showed nuclear positive staining and broader expression patterns across all cartilage layers in all groups (Fig. 2C). Extracellular matrix showed background staining in all groups likely due to the fact that the antibody utilized was made in a rabbit. Quantification of pTAK1 positive cells in the entire cartilage area showed that the Losartan group did not decrease pTAK1 expression compared to three other groups (Fig. 2F).

### 3.3 Effect of losartan on cancellous bone and cortical bone microarchitecture of the tibia

**Fig. 3:**
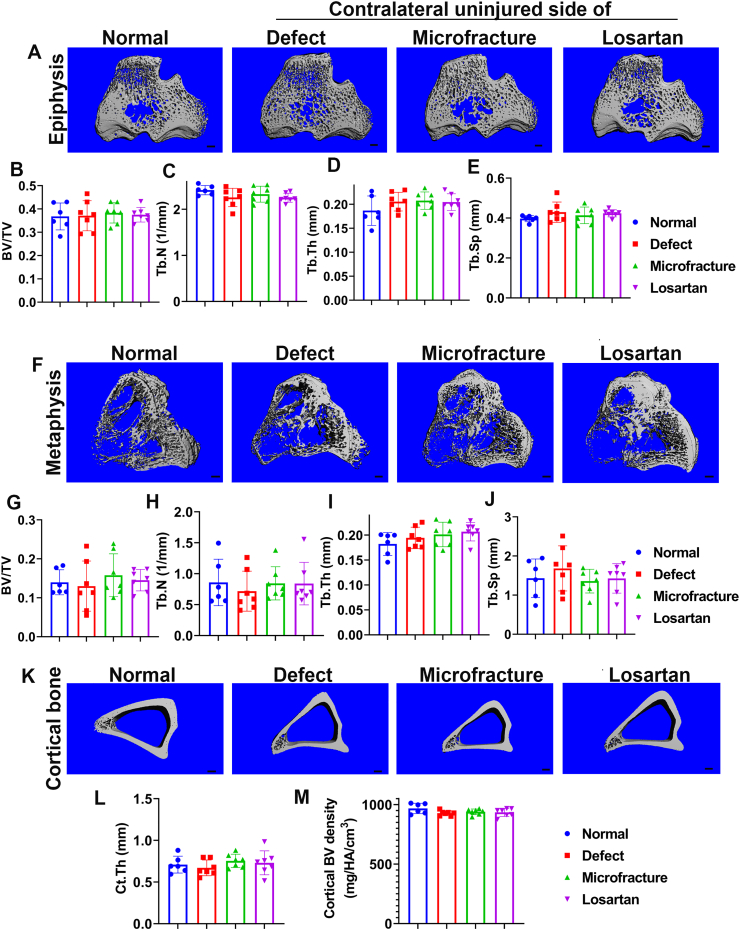
Micro-CT assessment of bone microarchitecture parameters of the tibia. (A) Top view of the epiphysis cancellous bone of tibia in each of the 4 groups. Similar and dense trabecular bone microarchitecture were observed in all groups. (B). BV/TV of epiphysis. (C) Tb.N of epiphysis. (D) Tb.Th of epiphysis. (E). Tb.Sp of epiphysis. No statistical difference was found for BV/TV, Tb.N, Tb.Th and Tb.Sp between the Losartan groups and any other groups. (F) Top views of the metaphysis cancellous bone of all groups. Sparse trabecular microarchitecture was shown among all groups compared to epiphysis. (G). BV/TV of metaphysis. (H). Tb.N of metaphysis. (I) Tb.Th of metaphysis. (J).Th.Sp of metaphysis. No statistical differences were found between the Losartan group and the other groups for BV/TV, BV density, Tb.N, Tb.Th and Tb.Sp in the metaphysis trabecular bone. (K) Top view of the midshaft tibial cortical bone. (L). Ct.Th of tibia cortical bone. (M). Cortical BV density. No statistical differences were found for the Ct.Th and BV density of cortical bone between Losartan group and the other three groups. Scale bars =1 mm for all images.

To investigate whether losartan treatment will affect bone microarchitecture, Micro-CT scanning was performed at different locations of the tibia. For the epiphysis, the top view of 3D reconstruction images from Micro-CT demonstrates highly dense trabecular bone in all groups and similar bone microarchitecture between the Losartan group and the other groups (Fig. 3A). No statistical differences were found for BV/TV, Tb.N, Tb.Th, or Tb.Sp of epiphyseal trabecular bone among the different groups (Fig. 3B-E). BV density of the epiphysis also showed no statistical differences between the Losartan group and the other groups (data not shown). Furthermore, the Micro-CT 3D images of metaphysis of all groups showed much fewer trabecular bone and similar microarchitecture between the Losartan group and the other groups (Fig. 3F). Quantification of the Micro-CT parameters showed no statistical differences between the Losartan group and the other groups for BV/TV, Tb.N, Tb.Th, Tb.Sp (Fig. 3G-J). BV density of the metaphysis also showed no statistical differences between the Losartan group and the other groups (data not shown). In addition, all groups showed similar structural morphology in the top view of the tibial cortical bone. Slight variations in the size of the bone were due to the animal size variations despite similar ages (Fig. 3K). Quantification of the cortical thickness (Ct. Th) and cortical BV density showed no statistical differences between the Losartan group and the other groups (Fig. 3 N—O).

### 3.4 Effect of losartan treatment on the histology of tibia cancellous bone

**Fig. 4:**
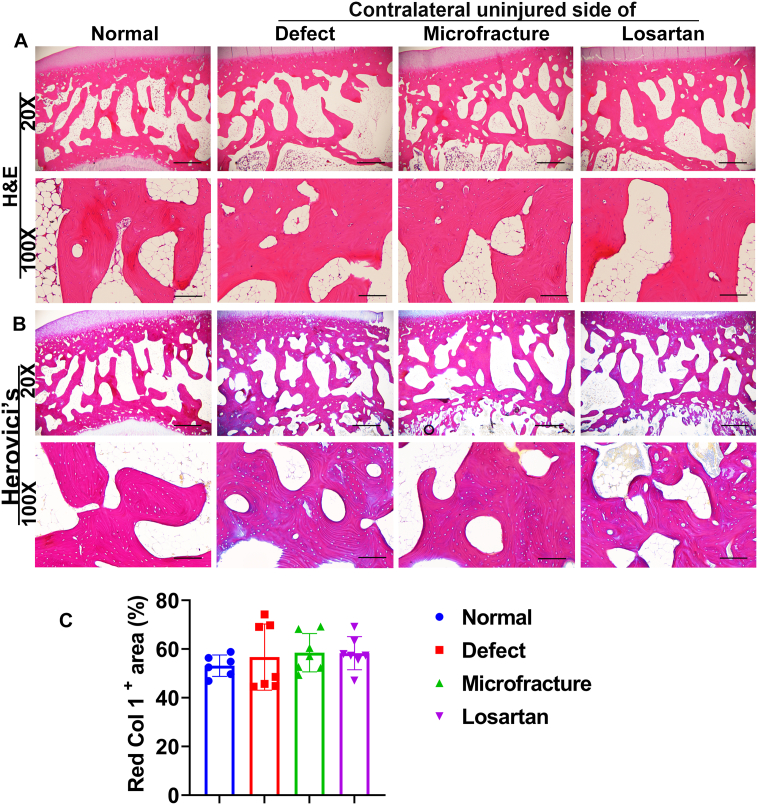
Histology assessment of the cancellous bone of the epiphysis. (A) H&E staining showed similar structure and general morphology of the cancellous bone of epiphysis between the Losartan group and the other groups at 20× magnification. No cell death and pathological changes were detected at 100× higher magnification in any groups. (B) Herovici's staining showed the Col 1 matrix in red in the trabecular bone of epiphysis. No structural changes in pink/red Col1 matrix at 20× and 100 × magnification. (C) No statistical differences were observed in Col1 area percentage in trabecular bone of epiphysis between Losartan group and other groups. Scale bars =1 mm for 20× and 200 μm for 100 × .

The epiphysis instead of metaphysis was chosen for analysis because the metaphysis has much fewer bone trabeculae. It is also very hard to get the exact comparable section level among specimens. H&E staining showed no differences in general morphology of the epiphysis cancellous bone between the Losartan group and the other groups, at lower magnification, and no changes in organization of osteocyte or osteoblasts at higher magnification. No abnormal cells death was found in the Losartan group compared to the other groups (Fig. 4A). Herovici's staining showed Col1 in red color and Col 3 in blue color. Staining intensity of red color Col1 was similar in the cancellous bone of epiphysis in all groups. No statistical differences were detected in Col1 matrix area percentages between the Losartan group and the other groups (Fig. 4B-C).

### 3.5 Effect of losartan on bone osteoblasts and other genes expression

**Fig. 5:**
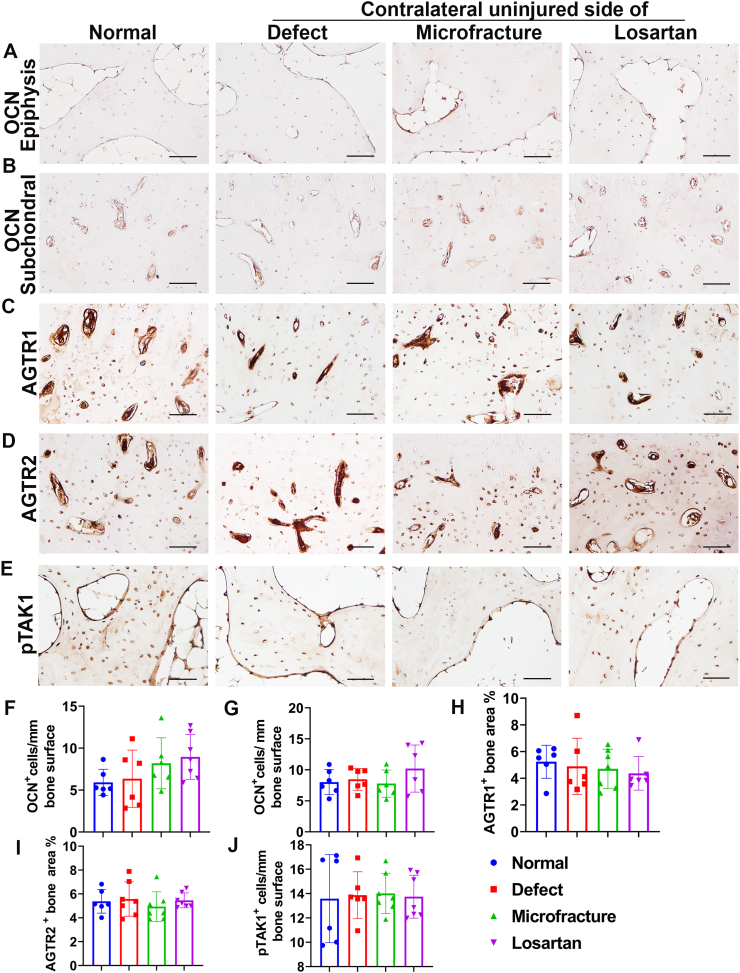
Immunohistochemistry staining of different genes of subchondral bone and cancellous bone of epiphysis of tibiae. (A and F) Immunohistochemistry of OCN and quantification of epiphysis cancellous bone. OCN positive cells showed sprout bud-like morphology on the bone surface in the cancellous bone of the epiphysis of tibia. No statistical difference of OCN positive osteoblasts on the bone surface was found between the Losartan group and the other groups. (B and G) OCN staining of subchondral bone and quantification. OCN positive cells located on bone surface of the trabeculae of subchondral bone. The Losartan group showed relatively higher OCN positive osteoblasts number than other groups, but this did not reach statistical significance. (C and H). Immunohistochemistry staining of AGTR1 and positive area percentage quantification. AGTR1 positive cells were seen on bone surface osteoblasts, osteocytes in the bone matrix as well as endothelial cells in the marrow cavity of subchondral bone. No statistical differences for AGTR1 positive area percentage were found between the Losartan group and the other groups. (D and I) Immunohistochemistry staining of AGTR2 of subchondral bone and quantification. AGTR2 positive cells located on bone surface osteoblasts, osteocytes embedding in the bone matrix and microvascular endothelial cells in bone marrow cavity. No statistical differences of AGTR2 positive area percentage were found between the Losartan group and the other groups. (E and J) pTAK1 expression and quantification in epiphysis. pTAK1 is expressed in the nuclei of bone surface osteoblasts and some osteocytes. Quantification of pTAK1 positive cells on bone surface showed no statistical differences between Losartan group and other groups. All images are 200×. Scale bars =100 μm for all images.

Next, it was investigated whether losartan treatment affects osteoblasts and gene expression in the cancellous bone of tibia. Immunohistochemistry staining of OCN was performed to reveal osteoblasts. Brown stained OCN positive cells are shown on the bone surface of cancellous bone of the epiphysis. OCN positive cells took the shape of sprouting bud (Fig. 5A). Quantification of the OCN positive osteoblasts demonstrated no statistical differences between the Losartan group and the other groups for epiphyseal trabecular bone (Fig. 5F). Furthermore, OCN positive osteoblasts also stained a brown color on the bone surface but showed flat morphology in subchondral bone in all groups (Fig. 5B). No statistical differences were found for the OCN positive osteoblasts between the Losartan group and the other group in the subchondral bone, but the Losartan group showed relatively higher OCN positive osteoblasts numbers than any other groups (Fig. 5G). To test if losartan treatment affects ATGR1 expression, immunohistochemistry staining for AGTR1 was performed with an analysis of the expression level on subchondral bone. AGTR1 was found to be expressed on the bone surface osteoblasts, osteocytes embedding in the bone matrix, and on the microvascular endothelial cells inside the bone marrow cavity as a brown color (Fig. 5C). Quantification of the AGTR1 expression area percentage showed no statistical differences between the Losartan group and the other groups despite the Losartan group showing relatively lower values (Fig. 5H). Similarly, AGTR2 is expressed on the bone surface osteoblasts, some osteocytes embedded in the bone matrix and microvascular endothelial cells in the marrow cavity of subchondral bone (Fig. 5D). Quantification of the AGTR2 positive area percentage indicated that the Losartan group did not have a significantly different AGTR2 expression level compared to other groups (Fig. 5I). Finally, immunohistochemistry of pTAK1 was also performed. pTAK1 positive cells were found to be mainly expressed on bone surface osteoblasts as shown in brown color in the nuclear. PTAK1 was also expressed in some osteocytes in the epiphysis trabecular bone of all groups (Fig. 5E). Quantification of pTAK1 positive cells on the bone surface of epiphysis indicated no significant statistical differences between the Losartan group and the other groups (Fig. 5J).

## 4 Discussion

This study investigated whether losartan effects the contralateral uninjured side's cartilage and bone homeostasis in the setting of using as a therapeutic for osteochondral defect and microfracture enhancement treatment. Oral losartan treatment was found to slightly increase Alcian Blue positive matrix and to decrease Col 3 in the articular cartilage with no significant effect on the expression levels of AGTR1, AGTR2 and pTAK1. Losartan treatment did not significantly change the cancellous and cortical bone architecture of normal uninjured side tibia as revealed by Micro-CT and histology. Losartan treatment also slightly increased OCN positive osteoblasts cells in epiphysis and subchondral cancellous bone. Losartan has no significant effects on AGTR1, AGTR2 and pTAK1 expression in cancellous bone. These results provide further safety evidence for using losartan as an microfracture augmentation therapeutic for cartilage repair in future human applications.

Microfracture has been used in clinical settings for many years to promote osteochondral defect healing, however, the regenerated tissue is mostly fibrocartilage rather than normal hyaline cartilage (Dai et al., 2014; Steadman et al., 2002; Xu et al., 2015). Previous work has demonstrated that oral administration of losartan enhanced cartilage repair by blocking TGF-β1 signaling pathways and fibrosis and thus resulting in a higher proportion of hyaline-like cartilage (Utsunomiya et al., 2020). Interestingly, this current study demonstrates that losartan slightly increases cartilage Alcian blue matrix and decreases Col 3 matrix in normal uninjured cartilage. Though small in content, Col3 is an indispensable component of the articular cartilage. It is primarily located on the surface of the articular cartilage in both normal and OA patients and is regarded as a marker of cartilage fibrosis which plays a detrimental role during cartilage repair (Aigner et al., 1993; Wotton and Duance, 1994; Young et al., 2000). These results demonstrated that losartan also has slight beneficial on normal cartilage while treating cartilage injury. Since losartan does not affect normal cartilage general morphology, therefore, it is safe to use as a microfracture augmentation therapy.

Several studies shown beneficial effect of losartan on cartilage injury via different mechanisms. It has been shown that losartan ameliorates adjuvant-induced arthritis in rats via down-regulation of AGTR1 and up-regulation of AGT2R expression (Wang et al., 2013). Others found that losartan attenuated the progression of degeneration in a mouse model of destabilization of the medial meniscus (DMM) induced OA via down-regulation of pSMAD2/3, a key signal molecule of TGFβ signaling pathways (Chen et al., 2015a). Additional studies have shown that losartan administration leads to a feedback loop that increases the content of free AngII, resulting in the activation of AngII type 2 receptor (AT2R) which exerts anti-inflammatory and anti-fibrotic effects (Habashi et al., 2011; Okada et al., 2006). However, it has also been shown that TGF-β1 signaling plays the opposite role by initiating and accelerating AC degeneration causing OA in adult joints (Bakker et al., 2001; Itayem et al., 1997). TAK1 is a down-stream enzyme of the TGF-β signaling pathway which has been shown to participate in the activation of the MAP kinase family in response to TGF-β stimulation (van Caam et al., 2017). Our previous study of injured osteochondral defect using the same set of rabbits showed oral losartan decreased TAK1 expression in the injured side fat pad and synovium (Utsunomiya et al., 2020). However, the current study demonstrated that oral losartan dosed at 10 mg/kg/day for 12 weeks did not significantly down regulate AGTR1, upregulate AGTR2 or change pTAK1 on normal cartilage likely due to the uninjured normal cartilage does not have abnormal expression of these genes. We did not perform pSMAD2/3 staining because the pSMAD 2/3 antibody for rabbits was not available. Hence, losartan does not affect normal cartilage signaling pathways, and, therefore, does not significantly interfere with normal cartilage homeostasis.

The effects of losartan on bone mass are controversial. Some studies shown angiotensin II receptors blockers (ARBs) might increase the risk of developing postmenopausal osteoporosis by blocking the TGF-β1 pathway, which has been shown to be important in bone mass maintenance (Akinci et al., 2007; Edwards et al., 2010; Faraji et al., 2016; Gazit et al., 1998). Others have shown that losartan improved bone density in hypertensive rats and increased both bone density and bone quality in normotensive rats using ovariectomized model of osteoporosis (Donmez et al., 2012; Shimizu et al., 2008). Another group explored the effects of losartan on bone deterioration in orchiectomized male hypertensive and normotensive rats and found a slight beneficial improvement in bone architecture in normotensive rats treated with high doses of losartan (25 mg/kg/day for 4 months), but no significant effects on bone mineral density (BMD), bone area, bone microarchitecture, and mechanical properties in both normotensive and hypertensive rats (Zhang et al., 2013). Further, it was found that long term treatment (3 months) with ARBs reduced serum TGF-β1 levels in ovariectomized rats but did not affect bone TGF-β1 content (Li et al., 2009). It was also reported that oral losartan (5 mg/kg/day) treatment in diabetic rats for 12 weeks did not reverse the decline in the BMD of diabetic bone as shown by dual-energy X-ray absorptiometry but recovered the mineral and collagen matrix properties of diabetic rat bone (Donmez et al., 2017). But losartan has a therapeutic effect on the physicochemical properties of diabetic bone and improved bone tensile strength at the material level (Donmez et al., 2017). Losartan also has been shown to increase bone mass and accelerate chondrocyte hypertrophy during skeletal development via down-regulation of osteoclast number and osteoclastogenesis in vitro by inhibition of the angiotensin- and RANK-induced phosphorylation of ERK1/2 pathway in mice (Chen et al., 2015b; Izu et al., 2009). The current study revealed that at the dose of 10 mg/kg/day, 12 weeks oral administration of losartan did not change tibial cancellous bone and cortical microarchitecture of contralateral uninjured side as demonstrated by Micro-CT as well general bone morphology and Col 1 bone matrix. Losartan treatment slightly increased OCN positive osteoblasts in the cancellous bone of both the epiphysis and subchondral bone. Hence, it is safe to use losartan to enhance microfracture-mediated cartilage repair.

The renin-angiotensin-aldosterone axis is known for its important role in cardiac physiology and electrolyte homeostasis (Zaman et al., 2002). This system also plays a role in bone metabolism (Izu et al., 2009). It has been shown AGTR2 receptor is expressed in both osteoblasts and osteoclasts in vitro. In addition, renin and angiotensin II-converting enzyme are expressed in bone cells in vivo. Treatment with the AGTR2 receptor blocker significantly enhanced bone mass via the enhancement of osteoblastic activity as well as the suppression of osteoclastic activity in vivo (Izu et al., 2009). AGTR1 may also serve as the mechanoreceptor of osteoblasts (Bandow et al., 2007). To investigate whether losartan treatment affects ATGR1, AGTR2 and pTAK1 in normal uninjured bone tissues during treatment of cartilage injury with losartan, immunohistochemistry of these three molecules was performed and demonstrated that AGTR1 and AGTR2 were all expressed on bone surface osteoblasts, some osteocytes as well as microvascular endothelial cells in the bone marrow cavity of subchondral bone. PTAK1 was expressed in bone surface osteoblasts and some osteocytes embedded in the bone matrix. Losartan treatment did not significantly change the expression level and pattern of these molecules. Thus, losartan does not significantly interfere with these signaling pathways in normal bone tissues.

## 5 Conclusion

In summary, oral losartan used as a microfracture augmentation therapeutic does not have significant effect on uninjured articular cartilage and bone based on our preclinical rabbit model. These results provided further evidence that the current regimen of using losartan as a microfracture augmentation therapeutic is safe with respect to bone and cartilage homeostasis and support clinical trials for its application in human cartilage repair.

## CRediT authorship contribution statement

JH, XG: Conceptualization; ZD: Investigation, methodology (Micro-CT and assist surgery and writing original draft; XG: investigation, methodology (all histology), formal analysis, software, validation, visualization, supervision and writing-draft original manuscript; HU: methodology (Surgery); JWA, JJR: Writing-reviewing and editing the manuscript; MH: Methodology (assist tissue harvest and lab support during revision); SR, project administration; MJP, resources, writing-revising the manuscript; JH: Funding acquisition, resources, supervision, writing-revising and final approval of the manuscript. All authors read and approved the manuscript.

## Declaration of competing interest

J.H received Royalties from Cooke Myocytes annually. M.J.P, Education payments from Linvatec (2015); speaking fees and consulting fees from Smith & Nephew (through 2018), Synthes GmbH (2019); royalties from DJO (2015/16), Linvatec (through 2018), and Smith & Nephew (2015/16); and hospitality payments from Siemens Medical Solutions (2016). All other authors have no competing interest to disclose.

## References

[bb0005] Aigner T., Bertling W., Stoss H., Weseloh G., von der Mark K. (1993). Independent expression of fibril-forming collagens I, II, and III in chondrocytes of human osteoarthritic cartilage. J. Clin. Invest..

[bb0010] Akinci B., Bayraktar F., Saklamaz A., Demir T., Yener S., Comlekci A., Ozcan M.A., Kebapcilar L., Yuksel F., Yesil S. (2007). Low transforming growth factor-beta1 serum levels in idiopathic male osteoporosis. J. Endocrinol. Investig..

[bb0015] Bakker A.C., van de Loo F.A., van Beuningen H.M., Sime P., van Lent P.L., van der Kraan P.M., Richards C.D., van den Berg W.B. (2001). Overexpression of active TGF-beta-1 in the murine knee joint: evidence for synovial-layer-dependent chondro-osteophyte formation. Osteoarthr. Cartil..

[bb0020] Bandow K., Nishikawa Y., Ohnishi T., Kakimoto K., Soejima K., Iwabuchi S., Kuroe K., Matsuguchi T. (2007). Low-intensity pulsed ultrasound (LIPUS) induces RANKL, MCP-1, and MIP-1beta expression in osteoblasts through the angiotensin II type 1 receptor. J. Cell. Physiol..

[bb0025] Bedair H.S., Karthikeyan T., Quintero A., Li Y., Huard J. (2008). Angiotensin II receptor blockade administered after injury improves muscle regeneration and decreases fibrosis in normal skeletal muscle. Am. J. Sports Med..

[bb0030] van Caam A., Madej W., Garcia de Vinuesa A., Goumans M.J., Ten Dijke P., Blaney Davidson E., van der Kraan P. (2017). TGFbeta1-induced SMAD2/3 and SMAD1/5 phosphorylation are both ALK5-kinase-dependent in primary chondrocytes and mediated by TAK1 kinase activity. Arthritis Res. Ther..

[bb0035] Chen R., Mian M., Fu M., Zhao J.Y., Yang L., Li Y., Xu L. (2015). Attenuation of the progression of articular cartilage degeneration by inhibition of TGF-beta1 signaling in a mouse model of osteoarthritis. Am. J. Pathol..

[bb0040] Chen S., Grover M., Sibai T., Black J., Rianon N., Rajagopal A., Munivez E., Bertin T., Dawson B., Chen Y., Jiang M.M., Lee B., Yang T., Bae Y. (2015). Losartan increases bone mass and accelerates chondrocyte hypertrophy in developing skeleton. Mol. Genet. Metab..

[bb0045] Chiu H.H., Wu M.H., Wang J.K., Lu C.W., Chiu S.N., Chen C.A., Lin M.T., Hu F.C. (2013). Losartan added to beta-blockade therapy for aortic root dilation in marfan syndrome: a randomized, open-label pilot study. Mayo Clin. Proc..

[bb0050] Dai L., He Z., Zhang X., Hu X., Yuan L., Qiang M., Zhu J., Shao Z., Zhou C., Ao Y. (2014). One-step repair for cartilage defects in a rabbit model: a technique combining the perforated decalcified cortical-cancellous bone matrix scaffold with microfracture. Am. J. Sports Med..

[bb0055] Deng Z., Gao X., Sun X., Amra S., Lu A., Cui Y., Eltzschig H.K., Lei G., Huard J. (2019). Characterization of articular cartilage homeostasis and the mechanism of superior cartilage regeneration of MRL/MpJ mice. FASEB J..

[bb0060] Donmez B.O., Ozdemir S., Sarikanat M., Yaras N., Koc P., Demir N., Karayalcin B., Oguz N. (2012). Effect of angiotensin II type 1 receptor blocker on osteoporotic rat femurs. Pharmacol. Rep..

[bb0065] Donmez B.O., Unal M., Ozdemir S., Ozturk N., Oguz N., Akkus O. (2017). Effects of losartan treatment on the physicochemical properties of diabetic rat bone. J. Bone Miner. Metab..

[bb0070] Edwards J.R., Nyman J.S., Lwin S.T., Moore M.M., Esparza J., O'Quinn E.C., Hart A.J., Biswas S., Patil C.A., Lonning S., Mahadevan-Jansen A., Mundy G.R. (2010). Inhibition of TGF-beta signaling by 1D11 antibody treatment increases bone mass and quality in vivo. J. Bone Miner. Res..

[bb0075] Faraji A., Abtahi S., Ghaderi A., Samsami Dehaghani A. (2016). Transforming growth factor beta1 (TGF-beta1) in the sera of postmenopausal osteoporotic females. Int. J. Endocrinol. Metab..

[bb0080] Farooq S., Leussink S., Sparrow L.M., Marchini M., Britz H.M., Manske S.L., Rolian C. (2017). Cortical and trabecular morphology is altered in the limb bones of mice artificially selected for faster skeletal growth. Sci. Rep..

[bb0085] Gao X., Usas A., Tang Y., Lu A., Tan J., Schneppendahl J., Kozemchak A.M., Wang B., Cummins J.H., Tuan R.S., Huard J. (2014). A comparison of bone regeneration with human mesenchymal stem cells and muscle-derived stem cells and the critical role of BMP. Biomaterials.

[bb0090] Gao X., Usas A., Lu A., Kozemchak A., Tang Y., Poddar M., Sun X., Cummins J.H., Huard J. (2016). Cyclooxygenase-2 deficiency impairs muscle-derived stem cell-mediated bone regeneration via cellular autonomous and non-autonomous mechanisms. Hum. Mol. Genet..

[bb0095] Gao X., Tang Y., Amra S., Sun X., Cui Y., Cheng H., Wang B., Huard J. (2019). Systemic investigation of bone and muscle abnormalities in dystrophin/utrophin double knockout mice during postnatal development and the mechanisms. Hum. Mol. Genet..

[bb0100] Gazit D., Zilberman Y., Ebner R., Kahn A. (1998). Bone loss (osteopenia) in old male mice results from diminished activity and availability of TGF-beta. J. Cell. Biochem..

[bb0105] Habashi J.P., Doyle J.J., Holm T.M., Aziz H., Schoenhoff F., Bedja D., Chen Y., Modiri A.N., Judge D.P., Dietz H.C. (2011). Angiotensin II type 2 receptor signaling attenuates aortic aneurysm in mice through ERK antagonism. Science.

[bb0110] Itayem R., Mengarelli-Widholm S., Hulth A., Reinholt F.P. (1997). Ultrastructural studies on the effect of transforming growth factor-beta 1 on rat articular cartilage. APMIS.

[bb0115] Izu Y., Mizoguchi F., Kawamata A., Hayata T., Nakamoto T., Nakashima K., Inagami T., Ezura Y., Noda M. (2009). Angiotensin II type 2 receptor blockade increases bone mass. J. Biol. Chem..

[bb0120] Keating G.M. (2009). Losartan/Hydrochlorothiazide: a review of its use in the treatment of hypertension and for stroke risk reduction in patients with hypertension and left ventricular hypertrophy. Drugs.

[bb0125] Kobayashi T., Uehara K., Ota S., Tobita K., Ambrosio F., Cummins J.H., Terada S., Fu F.H., Huard J. (2013). The timing of administration of a clinically relevant dose of losartan influences the healing process after contusion induced muscle injury. J. Appl. Physiol..

[bb0130] Kobayashi M., Ota S., Terada S., Kawakami Y., Otsuka T., Fu F.H., Huard J. (2016). The combined use of losartan and muscle-derived stem cells significantly improves the functional recovery of muscle in a young mouse model of contusion injuries. Am. J. Sports Med..

[bb0135] Laverty S., Girard C.A., Williams J.M., Hunziker E.B., Pritzker K.P. (2010). The OARSI histopathology initiative - recommendations for histological assessments of osteoarthritis in the rabbit. Osteoarthr. Cartil..

[bb0140] Li Y.Q., Ji H., Shen Y., Ding L.J., Zhuang P., Yang Y.L., Huang Q.J. (2009). Chronic treatment with angiotensin AT1 receptor antagonists reduced serum but not bone TGF-beta1 levels in ovariectomized rats. Can. J. Physiol. Pharmacol..

[bb0145] Logan C.A., Gao X., Utsunomiya H., Scibetta A.C., Talwar M., Ravuri S.K., Ruzbarsky J.J., Arner J.W., Zhu D., Lowe W.R., Philippon M.J., Huard J. (2021). The beneficial effect of an intra-articular injection of losartan on microfracture-mediated cartilage repair is dose dependent. Am. J. Sports Med..

[bb0150] McDonald M.A., Simpson S.H., Ezekowitz J.A., Gyenes G., Tsuyuki R.T. (2005). Angiotensin receptor blockers and risk of myocardial infarction: systematic review. BMJ.

[bb0155] Nakama G.Y., Gonzalez S., Matre P., Mu X., Whitney K.E., Utsunomiya H., Arner J.W., Philippon M.J., Ravuri S., Huard J. (2020). Effect of Oral losartan on orthobiologics: implications for platelet-rich plasma and bone marrow concentrate-a rabbit study. Int. J. Mol. Sci..

[bb0160] Okada H., Inoue T., Kikuta T., Watanabe Y., Kanno Y., Ban S., Sugaya T., Horiuchi M., Suzuki H. (2006). A possible anti-inflammatory role of angiotensin II type 2 receptor in immune-mediated glomerulonephritis during type 1 receptor blockade. Am. J. Pathol..

[bb0165] Rajkumar D.S., Faitelson A.V., Gudyrev O.S., Dubrovin G.M., Pokrovski M.V., Ivanov A.V. (2013). Comparative evaluation of enalapril and losartan in pharmacological correction of experimental osteoporosis and fractures of its background. J. Osteoporos..

[bb0170] Ravera M., Re M., Deferrari L., Vettoretti S., Deferrari G. (2006). Importance of blood pressure control in chronic kidney disease. J. Am. Soc. Nephrol..

[bb0175] Shimizu H., Nakagami H., Osako M.K., Hanayama R., Kunugiza Y., Kizawa T., Tomita T., Yoshikawa H., Ogihara T., Morishita R. (2008). Angiotensin II accelerates osteoporosis by activating osteoclasts. FASEB J..

[bb0180] Steadman J.R., Rodkey W.G., Rodrigo J.J. (2001). Microfracture: surgical technique and rehabilitation to treat chondral defects. Clin. Orthop. Relat. Res..

[bb0185] Steadman J.R., Rodkey W.G., Briggs K.K. (2002). Microfracture to treat full-thickness chondral defects: surgical technique, rehabilitation, and outcomes. J. Knee Surg..

[bb0190] Steadman J.R., Rodkey W.G., Briggs K.K. (2010). Microfracture: its history and experience of the developing surgeon. Cartilage.

[bb0195] Truong M.D., Chung J.Y., Kim Y.J., Jin L.H., Kim B.J., Choi B.H., Min B.H. (2014). Histomorphochemical comparison of microfracture as a first-line and a salvage procedure: is microfracture still a viable option for knee cartilage repair in a salvage situation?. J. Orthop. Res..

[bb0200] Turner N.J., Pezzone M.A., Brown B.N., Badylak S.F. (2013). Quantitative multispectral imaging of Herovici's polychrome for the assessment of collagen content and tissue remodelling. J. Tissue Eng. Regen. Med..

[bb0205] Utsunomiya H., Gao X., Deng Z., Cheng H., Nakama G., Scibetta A.C., Ravuri S.K., Goldman J.L., Lowe W.R., Rodkey W.G., Alliston T., Philippon M.J., Huard J. (2020). Biologically regulated marrow stimulation by blocking TGF-beta1 with losartan Oral administration results in hyaline-like cartilage repair: a rabbit osteochondral defect model. Am. J. Sports Med..

[bb0210] Vos M.B., Jin R., Konomi J.V., Cleeton R., Cruz J., Karpen S., Rodriguez D.S., Frediani J.K., McCracken C., Welsh J. (2018). A randomized, controlled, crossover pilot study of losartan for pediatric nonalcoholic fatty liver disease. Pilot Feasibility Stud..

[bb0215] Wang D., Hu S., Zhu J., Yuan J., Wu J., Zhou A., Wu Y., Zhao W., Huang Q., Chang Y., Wang Q., Sun W., Wei W. (2013). Angiotensin II type 2 receptor correlates with therapeutic effects of losartan in rats with adjuvant-induced arthritis. J. Cell. Mol. Med..

[bb0220] Wotton S.F., Duance V.C. (1994). Type III collagen in normal human articular cartilage. Histochem. J..

[bb0225] Xu X., Shi D., Shen Y., Xu Z., Dai J., Chen D., Teng H., Jiang Q. (2015). Full-thickness cartilage defects are repaired via a microfracture technique and intraarticular injection of the small-molecule compound kartogenin. Arthritis Res. Ther..

[bb0230] Yao X., Carleton S.M., Kettle A.D., Melander J., Phillips C.L., Wang Y. (2013). Gender-dependence of bone structure and properties in adult osteogenesis imperfecta murine model. Ann. Biomed. Eng..

[bb0235] Young R.D., Lawrence P.A., Duance V.C., Aigner T., Monaghan P. (2000). Immunolocalization of collagen types II and III in single fibrils of human articular cartilage. J. Histochem. Cytochem..

[bb0240] Zaman M.A., Oparil S., Calhoun D.A. (2002). Drugs targeting the renin-angiotensin-aldosterone system. Nat. Rev. Drug Discov..

[bb0245] Zhang Y.F., Qin L., Kwok T.C., Yeung B.H., Li G.D., Liu F. (2013). Effect of angiotensin II type I receptor blocker losartan on bone deterioration in orchiectomized male hypertensive and normotensive rats. Chin. Med. J..

